# Economic knowledge and the scientization of policy advice

**DOI:** 10.1007/s11077-018-9316-6

**Published:** 2018-04-05

**Authors:** Johan Christensen

**Affiliations:** Institute of Public Administration, Leiden University, PO Box 13228, 2501 EE Den Haag, The Netherlands

**Keywords:** Economics profession, Expertise, Knowledge, Policy advice, Policy advisory systems, Advisory commissions, Scientization

## Abstract

The growing role of economic expertise in contemporary policy-making has received increasing scholarly attention. Yet, so far, this discussion has only been tenuously linked to relevant debates in public policy and administration, such as the work on policy advisory systems. The article attempts to bridge this gap by examining the changing reliance on academic economic knowledge within policy advisory bodies. It does so by analysing appointments and citation patterns in Norwegian advisory commissions in economic policy over the last 45 years. The analysis shows a marked increase in the number of academic economists appointed to commissions and in citations to economic literature. Moreover, it reveals an orientation towards the most prestigious outlets of the international economics discipline. This development can be interpreted as a scientization of policy advice in the economic field, that is, a growing reliance on academic expertise for analysis and arguments about public policy.

## Introduction

There is a growing debate in the social sciences about the “scientification” or “expertization” of political life, that is, the increasing reliance on scientific experts and expertise in politics and policy-making (Weingart 1999; Turner 2003; Fischer 2009). Within this broader trend, the place of *economic* knowledge and reasoning in contemporary policy-making has received particular attention (Markoff and Montecinos 1993; Fourcade 2006, 2009; Hirschman and Berman 2014). Studies have pointed to the growing influence of economics in government and society and the increasing tendency to look to economists for answers to all kinds of policy problems.

However, this discussion has only been tenuously linked to relevant debates in public policy and administration. The mostly sociological literature about economists has shied away from specific questions related to the decision-making process, policy advice, political-administrative relationships, etc. Conversely, relevant literature in public policy and administration has paid little systematic attention to the changing role of academic expertise in policy-making. This includes the literature on “policy advisory systems”, which examines the range of actors and organizations that provide decision-makers with policy-relevant knowledge and arguments (Craft and Howlett 2013; Craft and Halligan 2017). This literature points to externalization—the growing reliance on advice from outside the public service—and politicization as the two predominant trends in policy advice in recent decades. By contrast, changes in the role of academic knowledge in policy advice have not been systematically addressed theoretically or empirically.

The article attempts to bridge this gap. Drawing on theoretical arguments about economic knowledge as a source of authority in policy-making, it points to “scientization” as a third possible trend in advisory systems. Scientization of policy advice is understood as the increasing reliance of decision-makers on policy advice provided by academic actors and based on scientific knowledge and claims. Empirically, the article examines the changing reliance on academic economic expertise in policy advisory bodies over time. This is investigated in the context of advisory commissions in Norway—a political system with strong statist and neo-corporatist traditions. While existing research on policy advisory systems centres on Westminster systems (Craft and Halligan 2017), recent studies have explored the particular patterns of policy advice in consensus-driven, neo-corporatist systems (e.g. Van den Berg 2017). It has been argued that scientific expertise is less likely to break through in neo-corporatist settings, given the central position of interest groups and the non-scientific knowledge and arguments that they provide (Blum et al. 2017). Norway can therefore be seen as an unlikely case for the growing reliance on academic knowledge in policy advice.

The article poses the following research question: *How did the reliance on academic economists and academic economic knowledge in Norwegian* ad hoc *advisory commissions in economic policy change during the period 1967*–*2013?* This question is analysed based on quantitative data on developments along two dimensions: appointments of academic economists to commissions and citations to economic literature in commission reports. Whereas appointments may indicate to what extent economists are regarded as legitimate and authoritative participants in discussions about public policy, citations provide information about whether policy arguments are justified in scientific terms and what kind of knowledge they are based on. These indicators do not fully or perfectly measure the reliance on academic economic knowledge. Yet, they constitute theoretically meaningful measures that capture key aspects of the concept and allow for comparison across a large number of units over time.

The analysis is based on a new dataset that includes all Norwegian advisory commissions on economic policy appointed in the period 1967–2013—a total of 80 commissions with altogether nearly 800 members and 4000 citations. The analysis shows a marked increase in the number of academic economists appointed to commissions, in particular as commission chairmen, and a steep rise in the number of citations to economic literature in commission reports. Moreover, the citation analysis reveals a strong orientation towards high-prestige international economic knowledge. The article argues that these developments can be interpreted as evidence for the scientization of policy advice in the economic field.

The article proceeds as follows: It first presents theoretical arguments about the growing role of economic knowledge in contemporary policy-making, before discussing the literature on policy advisory systems and proposing scientization as a third trend in policy advice. After describing the Norwegian case and the research design, the article traces developments in commission appointments and citations and explores the relationship between the two. In the final sections, the results are discussed and broader implications are drawn.

## Economic knowledge as a source of authority in policy-making

Several observers point to the growing reliance on scientific experts and knowledge in politics and policy-making as a defining feature of contemporary governance, referring to this phenomenon as the “scientification” (Weingart 1999) or “expertization” (Turner 2003) of politics. Academic knowledge—that is, abstract knowledge generated within academic disciplines and acquired through extensive training—has become an increasingly important source of legitimacy in the political sphere, challenging traditional sources of political legitimacy and existing ways of making decisions. Examples of this trend are the increasing power and autonomy of expert bodies like courts, regulatory agencies, central banks and international financial institutions (Babb 2003; Vibert 2007), the movement towards “evidence-based” policy-making (Nutley et al. 2007), and the growing need to back up political arguments with knowledge and research (Weingart 1999).

The place of *economic* expertise and reasoning in contemporary policy-making has received particular attention. There is by now a substantial body of the mostly sociological literature about the influence of economics on political decision-making at different levels of governance and in a range of national contexts (Markoff and Montecinos 1993; Babb 2004; Fourcade 2006, 2009; Chwieroth 2009; Reay 2012; Hirschman and Berman 2014). This literature points to the increasing authority granted to economists in discussions about how to organize economy and society. Economic knowledge has come to be perceived as a legitimate source of arguments about all kinds of policy problems, and indeed, as a *more* legitimate source of arguments than other forms of knowledge (Hirschman and Berman 2014).

The peculiar influence of economics has been ascribed to two main dynamics. On the one hand, economists possess knowledge that is seen by governments as crucial for ensuring prosperity and growth (Fourcade 2006). Nation-states have become increasingly concerned with governing the economy and boosting economic performance. As a result, the special knowledge of economists about how economies work has become instrumental to government activity. But the reliance on economic knowledge also has a more ceremonial side (Markoff and Montecinos 1993). Displaying expertise in economic matters has become a way of showing that policy-making is sound, rational and responsible. Economic experts and expertise are used to increase legitimacy, by infusing policy-making with an appearance of rationality (see Meyer and Rowan 1977). This dual motive for the use of economic expertise mirrors the more general distinction between the instrumental and legitimating functions of expert knowledge in policy-making (Weiss 1979; Boswell 2008; Weible 2008).

Yet, economic knowledge also has some specific characteristics that sets it apart from other types of academic knowledge. The first is its *transnational* character. Economics has become a truly global profession where ideas, practices and authority flow freely across national borders, from the “core” of top US economics departments to the “periphery” of economists working in universities, governments and businesses around the world (Fourcade 2006). In this transnational field, the international discipline is a primary point of reference and ties to the international profession are as important as linkages to the national profession. Indeed, links to the international economic profession may be used as weapons in local struggles for intellectual jurisdiction over a particular domain. “Connections to (mainly) US-based standards of work and professional practice”, argues Fourcade, “are routinely used in the local competition whereby different professional segments and groups seek to assert their authority on particular jurisdictions (scientific, corporate, or political)” (Fourcade 2006, 145; see also Dezalay and Garth 2002). In other words, actors may seek to legitimate arguments in the political sphere by making reference to international economic knowledge.

A second and related characteristic of the economics field is its *hierarchical* character. Compared to other disciplines, “economics looks both more inward and towards the top of its internal hierarchy” (Fourcade et al. 2015, 96). Economic knowledge production is organized according to a clearly perceived hierarchy, with a handful of US economics departments and journals perched at the top. Analyses of citation and recruitment patterns show that these leading institutions have extraordinary authority over the rest of the field (Fourcade et al. 2015). The implication is that intellectual prestige in economics is defined strictly with respect to this internal status hierarchy. Degrees from leading economics departments or publications in top-tier economics journals constitute the supreme sources of authority over economic matters.

The expanding role of economic knowledge in policy-making has taken different forms. One is the increasing power and autonomy of economic expert institutions at the international and national levels, such as the International Monetary Fund (IMF) (Babb 2003; Chwieroth 2009) or European and national central banks (Marcussen 2006). Another expression is the rise of economists within public bureaucracies (Christensen 2017) and, in some cases, also into higher political offices such as minister of finance or president (Markoff and Montecinos 1993; Babb 2004). Furthermore, scholars point to how economics shapes the “cognitive infrastructure of policy-making” through the diffusion of economic measures like GDP or decision-making tools such as cost–benefit analysis (see Hirschman and Berman 2014).

A final mode of influence is the use of economic knowledge in policy advice bodies. Scholars have started to address this theme, most prominently by mapping different national “knowledge regimes” in economic policy, that is, “the organizational and institutional machinery that generates data, research, policy recommendations, and other ideas that influence public debate and policymaking” (Campbell and Pedersen 2014, 3). However, this work focuses explicitly on applied policy research organizations rather than on academic economic knowledge and the impact of professional dynamics (see Campbell and Pedersen 2015, 682–84). Moreover, its account of changes in knowledge regimes is based largely on qualitative interviews with involved actors rather than on quantitative data. Yet, more fundamentally, what is missing in the work on knowledge regimes—and in the literature on the power of economics more generally—is engagement with related discussions in public policy and administration. As a consequence, the implications of arguments about the growing reliance on economic knowledge for questions related to policy advice and the decision-making process remain unexplored. The next section looks more closely at the literature on policy advisory systems and its treatment of academic knowledge.

## Policy advisory systems and academic knowledge

Policy advice institutions have received increasing scholarly attention in recent years, particularly in the public policy literature about “policy advisory systems” (Craft and Howlett 2013; Craft and Halligan 2017). This work examines the system of actors and organizations that provide decision-makers with policy advice, that is, “analysis of problems and the proposing of solutions” (Halligan 1998, 1686). This ranges from permanent civil servants and government research agencies to think tanks and consultants. Suppliers of policy advice are classified according to their location (inside or outside the public service) and the degree of government control over advice (Halligan 1995; Craft and Halligan 2017).

A central issue in this literature is how policy advisory systems have changed in the context of broader developments in politics, administration and society (Hustedt and Veit 2017). Scholars have pointed to two predominant trends. The first is the *externalization* of the provision of policy advice from the public service to a multitude of advisory actors outside government, such as think tanks or consultancy firms. The second is the *politicization* of policy advice, that is, the growing use of partisan political advisers and greater political steering of the policy formulation process (Craft and Howlett 2013).

This literature has paid less attention to the changing role of academic knowledge in policy advice. Although the question of knowledge utilization is key to the whole endeavour of studying policy advisory systems (Hustedt and Veit 2017, 42; Craft and Halligan 2017), this work has little to say about how the involvement of academic experts and expertise in advice-giving has developed over time. Some scholars mention “scientization” as one of the exogenous processes that may have affected policy advisory systems (Veit et al. 2017, 88–89), and others present results that suggest a greater reliance on universities and research institutes for advice (Van den Berg 2017). Yet, this literature does not provide theoretical concepts for understanding the evolving role of academic knowledge within advice institutions. Developments along this axis do not fit neatly into the existing analytical scheme based on the location of advice and the degree of governmental control.

The theoretical arguments presented in the previous section point to *scientization* as a third possible trend in advisory systems. Scientization of policy advice can be understood as the increasing reliance of decision-makers on academic expertise for advice about public policy. Scientization implies a change in who provides advice: analysis and arguments about policy are increasingly supplied by academic actors. But it also entails a shift in the basis for policy advice: advice is increasingly based on scientific knowledge and claims. That is, arguments about policy not only draw on academic knowledge but are also justified in scientific terms. To be sure, this does not necessarily mean that this policy advice comes closer to the “truth” than other advice. Presenting an argument as scientific can be a deliberate strategy to increase its credibility and authority (e.g. Marcussen 2006).

It can be objected that scientization is simply a form of externalization. Yet, scientization refers to a more specific phenomenon, since it distinguishes academics from external advisory actors oriented towards advocacy, such as interest groups (Blum and Brans 2017). Whether policy advice is provided by a business association or a university researcher is arguably an important distinction. Scientization may also overlap with politicization if scientific knowledge is used to legitimate rather than inform policy-making. Yet, there are obvious conflicts between the two notions: political steering runs counter to scientific ideals of independence and objectivity, and the use of partisan political advisors conflicts with the reliance on academic advisors. Scientization thus provides a useful addition to existing concepts for understanding changes in policy advisory systems.

The article explores the scientization of policy advice by looking at the changing reliance on academic economic knowledge in advisory bodies. To be sure, changes in the use of *economic* knowledge are not necessarily representative of academic knowledge in general. However, economic knowledge constitutes an especially useful case for assessing scientization. First of all, economics emerged in the post-war era as a general technique of government, with proclaimed relevance for a whole range of policy areas. This sets it apart from sciences such as medicine or chemistry, whose application is limited to particular policy areas. Therefore, analysing the reliance on economic knowledge can say something about changes in the broader business of governing. Second, economics is a discipline whose scientific status is contested. The economics discipline has sought to establish itself as a “real science” through extensive formalization, that is, the formal modelling of theoretical arguments based on advanced mathematics (Fourcade 2009, chap. 2). This approach has been used to claim that economic analysis produces scientific conclusions. Examining scientization in the case of economic knowledge thus allows us to tap into the dynamics surrounding the presentation of scientific claims by professional groups and the acceptance of these claims by decision-makers (see Marcussen 2006).

Based on the theoretical arguments discussed in the previous section, we develop the following expectations about changes in economic policy advice. First, we expect an increase in the appointment of academic economists to policy advisory bodies. Second, we expect policy advisory bodies to draw increasingly on academic economic knowledge and scientific claims in their work. Third, and more specifically, we expect policy advisory bodies to draw increasingly on academic knowledge from the top of the international economics discipline.

## Advisory commissions in Norway

The theoretical expectations about the changing reliance on academic knowledge in policy advice are examined in the empirical context of Norway—a political system with strong statist and neo-corporatist features. The Scandinavian countries are often described as “strong states” where the government bureaucracy traditionally has played a leading role in the development of public policies (Heclo 1974; Lindvall and Rothstein 2006). Moreover, organized interests have traditionally participated extensively and regularly in the preparation and implementation of policy (Blom-Hansen 2000; Christiansen et al. 2010). These features are reflected in the Norwegian policy advisory system. Norwegian advisory boards and commissions have been regarded primarily either as venues for the institutionalized involvement of interest groups in policy-making (Christiansen et al. 2010; Rommetvedt et al. 2012) or as bodies subject to extensive government control and with a strong civil service presence (Nordby 1999).

Much existing research on policy advisory systems has focused on Westminster systems (see Craft and Halligan 2017). Yet, the distinct dynamics of policy advice in countries with consensus-based politics and neo-corporatist traditions have received more attention recently (Van den Berg 2017). It has been argued that scientific knowledge plays a less important role in neo-corporatist settings. Given the central position of interest groups, the information and arguments provided by stakeholders will be equally or more relevant that scientific expertise, and academics will have difficulties securing access to an already crowded policy advice field (Blum et al. 2017). The Norwegian system is therefore *not* a likely case for the growing reliance on academic knowledge in policy advice. Rather, it is a case where the use of scientific expertise stands up against important competing concerns about interest representation and state control in policy advice.

The article examines a specific type of Norwegian advice bodies, namely official ad hoc advisory commissions. These commissions are appointed by Cabinet or a ministry to investigate a specific policy issue and propose solutions. Their members are drawn from the civil service, interest groups, academia, political parties, private companies or other fields. Commissions synthesize existing knowledge and sometimes also carry out or commission new research. Their analysis and recommendations are presented in a report that is submitted to the relevant ministry and made publically available. This advice usually feeds into the early stages of the decision-making process, that is, before concrete policies are proposed by the government.

The rationale for examining ad hoc advisory commissions is their central place in the Norwegian policy advisory system. Commissions have traditionally been seen as a vital part of the decision-making process on major policy issues (Arter 2008). On average, more than 30 commissions were appointed annually in the period 1967–2013, making the Norwegian commission system far more extensive than for instance the system of royal commissions in the Westminster countries (see Craft and Halligan 2017). Moreover, existing studies show that these commissions can have a significant influence on policy and policy debates. For instance, Lie and Venneslan (2010) and Christensen (2017) document how commissions provided the blueprint for a series of economic reforms from the 1980s onwards, and Tellmann (2016) finds that commissions had an important impact on thinking around climate change policy.

## Data and methods

The article investigates the changing role of academic economic knowledge in policy advice through a quantitative analysis of Norwegian ad hoc advisory commissions. The analysis includes all commissions in the economic policy area that had a policy-preparing function and were appointed in the period 1967–2013 (see “Appendix 1” for details on data and coding). This encompasses commissions on topics such as taxation, wage policy, pensions, financial regulation and budgeting. The analysis comprises 80 commissions with a total of 779 members and 3936 citations.

The reliance on academic economic knowledge is traced along two dimensions. The first is the appointment of academic economists as commission members and chairmen. “Academic economists” are defined as economists working at universities and research institutes. In Norway, members of commissions are appointed by the responsible ministry. The appointment of academic economists as commission members indicates to what extent their knowledge is seen as useful, relevant or legitimate in discussions about a given policy issue. The ministry also appoints a chairman to oversee and organize the work of the commission and to represent the commission vis-à-vis the authorities and the public. The position as chairman arguably has a ceremonial side, given that he or she is the guarantor for the integrity and soundness of the commission’s work and the most visible representative of the commission. Indeed, Norwegian commissions are often known simply by the name of the chairman. The appointment of academic economists as commission chairmen may therefore provide an indication of the more symbolic use of economic expertise.

The second dimension is the use of citations to academic economic knowledge in commission reports. The citation analysis is frequently used to examine the dynamics of knowledge production (e.g. Uzzi et al. 2013) and the interaction within and between academic disciplines (e.g. Fourcade et al. 2015). Yet, it can also be employed to analyse the relationship between economic science and policy-making. In general, citations link an argument to existing principles, practice or knowledge. These links have both an instrumental function and a legitimizing function. On the one hand, they provide information about what kind of knowledge the argument is based on. On the other hand, they can be used to lend credibility and authority to an argument, e.g. by indicating that it is based on scientific knowledge. This double function must be taken into account when analysing citation data.

In the article, the number of citations to different sources of economic knowledge is examined to say something about what kind of knowledge policy advice is based on and to what extent advice is justified with reference to scientific knowledge. Some caveats are in order. First, a citation does not necessarily indicate support for the knowledge or principle cited; citations can also be used to counter or de-legitimize the source. Second, some of the cited sources will have greater influence on the content than others, something that a citation count does not pick up. Yet, like the reference list of an academic article provides a rather good indication of the ideas contained in the article, the literature cited in a commission report can give us a sense of the dominant themes and arguments in the report. As such, citations offer a theoretically meaningful measure of the reliance on academic knowledge, which allows for comparison across a large number of commissions and over time.

Finally, the article examines the relationship between these two dimensions. Using ordinary least squares (OLS) regression, it looks at the effect of the presence of academic economists as commission members and chairman, respectively, on the use of citations to academic economic literature. (The regression analysis is described in more detail below.)

## The changing role of economic knowledge in Norwegian advisory commissions

**Fig. 1:**
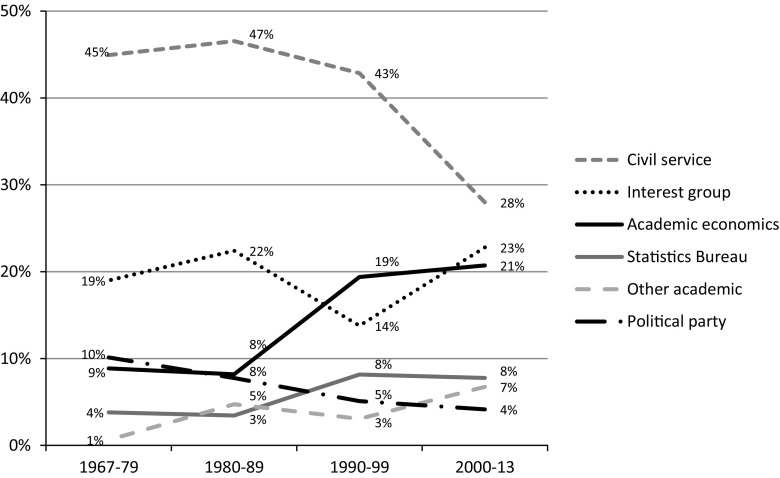
Affiliation of commission members. Percent of all members. *Note*: commissions are categorized according to year of appointment

Looking first at the number of academic economists appointed to commissions in economic policy, Fig. 1 displays the distribution of commission members (including chairmen but excluding secretariats) by affiliation during different periods.

The figure shows that the share of academic economists appointed to commissions increased markedly over time, from less than 10% of members in the 1970s and 1980s to around 20% in the 1990s and 2000s.^1^ The share of members from a related category—researchers from the Norwegian statistics bureau—also increased slightly during this period, accounting for another 8% of commission members in the 1990s and 2000s. By contrast, the portion of members drawn from the civil service dropped, from nearly half of all members in the 1970s, 1980s and 1990s to less than 30% in the 2000s. The share of commission members from the political parties also declined, from 10% in the 1970s to only 4% in the 2000s. We also see that interest groups retained a substantial share of commission posts.

The changes in appointments are even stronger if we look at the background of commission chairmen (see Table 1).

**Table 1:** Affiliation of commission chairmen. Absolute numbers

	Civil service	Interest group	Academic economics	Other academic	Statistics bureau	Political party	Other	Total
1967–1979	7	0	3	0	2	3	1	16
1980–1989	10	0	1	6	1	1	2	21
1990–1999	6	0	12	0	3	0	2	23
2000–2013	2	1	8	3	5	0	1	20

We see a growing tendency to appoint academic economists as commission chairmen. While economics professors and researchers accounted for little more than one in ten chairmen before 1990, they headed nearly half of all commissions after 1990. On top of this, a number of commission heads in the 1990s and 2000s were drawn from the statistics bureau. By contract, the number of civil servants appointed to lead commissions declined from nearly half of all chairmen before 1990 to less than one in five chairmen after 1990. The number of commissions headed by politicians also dropped to zero in the period after 1990. Interestingly, the appointment of an academic economist as commission chairman was *not* positively correlated with the share of academic economists among the other members of the commission.^2^ This may indicate that the reliance on academic economists as commission members and chairmen, respectively, reflected different underlying rationales.

**Fig. 2:**
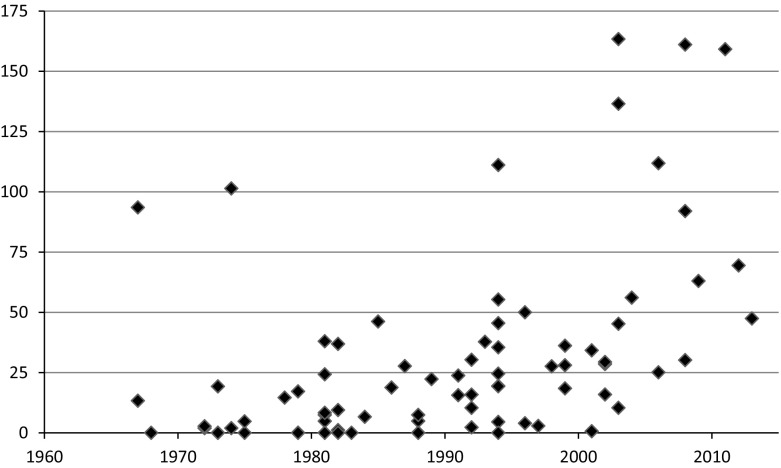
Citations in commission reports, per 100 pages. Each data point represents one report

Beyond the participation of economists, the use of citations in commission reports provides information about the extent to which economic knowledge is drawn upon and the type of economic knowledge that is used. Looking first at the overall use of citations, Fig. 2 shows the citation frequency in commission reports over time, including all types of citations.

As we see, the use of citations in commission reports has increased sharply over time. While many reports from the 1970s and 1980s contained no citations at all, all reports from the last 10 years had at least 25 citations per 100 pages and a handful contained more than 100 citations per 100 pages. In other words, it has become increasingly common to make explicit reference to existing knowledge, principles and considerations in commission reports.

**Fig. 3:**
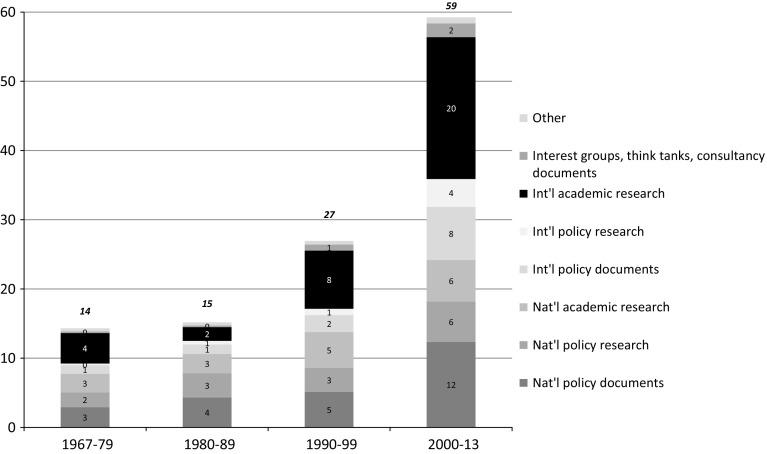
Average number of citations in commission reports, per 100 pages of report. By type of source

To what extent were the citations in commission reports to academic knowledge? Figure 3 shows the frequency of citations to different sources over time (see “Appendix 1” for details on coding).

Remarkably, the largest number of citations was to international academic research. International academic research was cited on average 20 times per 100 pages in reports from commissions appointed after 2000—a fivefold increase from the 1970s. The majority of these citations were to articles in international scientific journals (discussed in more detail below), while the rest were to international academic books, book chapters and working papers. There was also a considerable number of citations to domestic academic research at the beginning of the period. But references to this type of knowledge increased slowly over time—from 3 citations per 100 pages in the 1970s and 1980s to 6 in the 2000s. More policy-oriented research was also cited in commission reports: domestic policy research (e.g. from the statistics bureau or the central bank) was an important source of citations throughout the period, and international policy research (i.e. research carried out by international organizations and foreign government bodies) was frequently cited in the 2000s. At the same time, a large number of citations were to domestic policy documents, such as existing commission reports, government white papers and bills submitted to parliament. By contrast, documents published by interest groups, think tanks and consultancy firms were rarely cited in commission reports. Whereas the distribution of citations across the major categories remained relatively stable over time, there was a certain shift towards international sources.

**Fig. 4:**
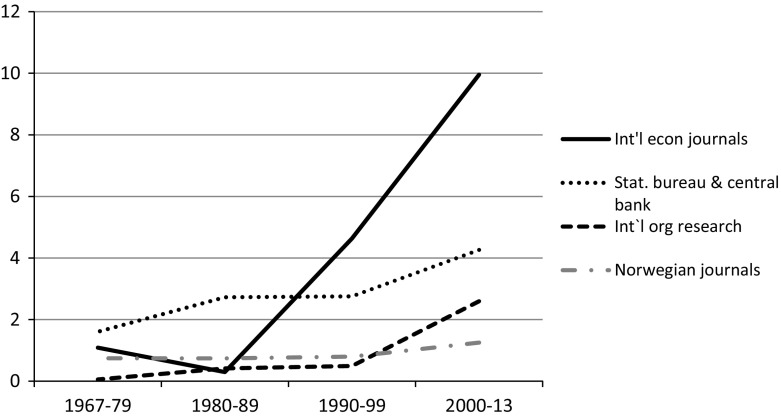
Number of citations to economic research, per 100 pages of report. Selected categories

If we look more closely at citations to *economic research*, the shift towards international sources comes more clearly into view. Figure 4 displays the number of citations to selected categories of economic research, ranging from policy-oriented domestic economic research to international economics journals.

The figure shows an exponential increase in the number of citations to international economics journals, which eclipsed all other sources of economic knowledge. Although citations to economic research from international organizations, such as the OECD, the IMF or the EU/ECB, also grew rapidly in the 2000s, this remained a much less important source of knowledge. Citations to domestic economic research—research issued by the Norwegian statistical bureau and central bank or published in Norwegian journals—also increased over time, but at a much slower pace. In other words, the economic knowledge cited in commission reports was increasingly of the international and highly scientific kind featured in international peer-reviewed journals.

**Fig. 5:**
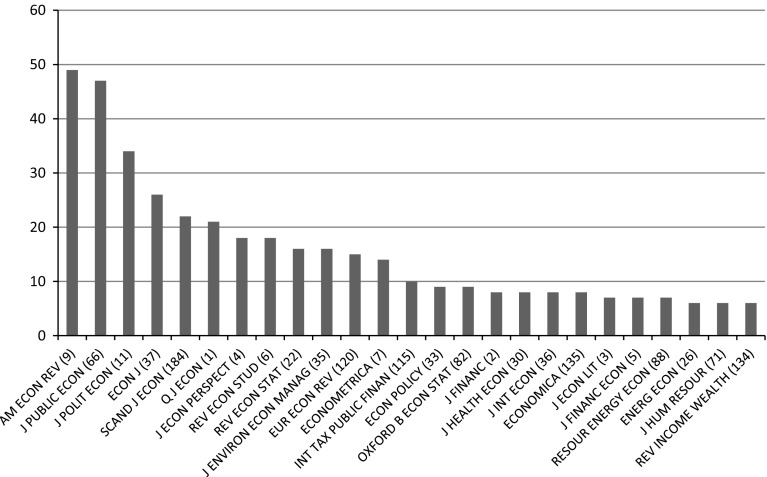
Most cited economics journals in commission reports. Journal ranking in parentheses

A further indication of the character of the economic knowledge used in commission reports is the profile of the economics journals cited. Figure 5 displays the international economics journals most frequently cited in commission reports, with the number in parentheses behind the journal name indicating the international ranking of the journal (according to the Thomson Reuters Journal Citations Report 2014).

Most striking is the predominance of citations to the very top journals of the economics discipline. The most cited journal in Norwegian commission reports was the *American Economic Review*, the flagship journal of the American economics profession. Other top-ranked journals such as the *Journal of Political Economy*, the *Quarterly Journal of Economics* and the *Review of Economic Studies* were also among the ten most cited journals in commission reports. In fact, the 25 journals most frequently cited by commissions included eight of the ten most prestigious journals in the economics field.

How was the large number of citations to high-prestige international economic research related to the appointment of economists to commissions? To examine this, the data on appointments and citations are combined in an OLS regression analysis. The dependent variable in the analysis is the number of citations to international economics journals per 100 pages of report, which is an appropriate measure of the reliance on top international economic knowledge.^3^ The independent variables of interest are the share of commission members who are academic economists (excluding chairmen) and whether the chairman is an academic economist. To control for other features of commission composition that may influence the use of citations, the share of commission members from the statistics bureau and whether the chairman is from the statistics bureau are included as control variables. Finally, to check for differences across periods in the use of citations, year of appointment is included as a control variable (see “Appendix 2” for descriptive statistics and correlations). The results of the analysis are shown in Table 2.

**Table 2:** OLS estimates of determinants of citations to international economics journals

Dependent variable: number of citations to international economics journals per 100 pages (log)				
	(1)	(2)	(3)	(4)
	*b* (se)	*b* (se)	*b* (se)	*b* (se)
Share of members academic economists	4.091*** (0.566)	4.211*** (0.560)	3.573*** (0.630)	2.986*** (0.595)
Chairman academic economist		0.418 (0.215)	0.523* (0.230)	0.238 (0.222)
Share of members statistics bureau			0.489 (1.291)	0.310 (1.182)
Chairman statistics bureau			0.817** (0.308)	0.608* (0.287)
Year of appointment (1967 = 0)				0.033*** (0.008)
Intercept	0.241 (0.127)	0.098 (0.145)	0.011 (0.145)	− 0.540** (0.193)
Adjusted *R*^2^	0.396	0.417	0.453	0.543
*N*	79	79	79	79

*p < 0.05; **p < 0.01; ***p < 0.001

We see that the share of academic economists among the commission members on its own has a positive and significant effect on the number of citations to international economics journals. This effect remains significant at the 0.001 level also after introducing controls. By contrast, having an academic economist as commission chairman does not have a significant effect on the volume of citations to international economics journals, except for in model 3. Of the control variables, having a chairman from the statistics bureau has a positive and significant effect on citations (whereas the share of members from the statistics agency does not), and year of appointment also has a positive and significant effect on citation frequency. The adjusted R-squared scores indicate that the variables included in the models account for a large share of the variance on the dependent variable. The share of academic economists among commission members on its own accounts for nearly 40% of the variance, whereas the full set of variables accounts for 54%. The analysis thus shows that citations to international economics journals in commission reports to a large extent were predicted by the participation of academic economists as commission members and not as chairmen.

## Discussion

What do the empirical results tell us about the changing reliance on academic economic expertise in Norwegian policy advisory bodies? First of all, the analysis shows a marked increase in the appointment of academic actors to commissions relative to other groups. The share of academic economists among members doubled over time. Their share of chairmen increased even more sharply: the important and highly visible chairman position was increasingly given to individuals with epistemic authority in the field of economics rather than to people with administrative or political authority. This did not mean that non-academic actors disappeared from the commissions. Although the share of civil servants on commissions dropped, they were still the largest group of members. And the proportion of interest groups representatives remained substantial. Yet, overall, the changes in appointments suggest a shift in who is regarded as legitimate providers of analysis and advice on economic policy issues, away from public officials and towards academic economists (cf. Hirschman and Berman 2014).

An important question is whether it makes sense to draw a sharp distinction between academic and bureaucratic actors in a context where most civil servants hold degrees in economics. One could see the relationship between the two groups more as an alliance based on a shared professional background and worldview. There are, however, good reasons to distinguish between the two groups. First, bureaucrats also have other loyalties, both to the political leadership and to their department. In Norway, civil servants may be instructed by the minister to take a certain stance within the commission, whereas academics act as independent experts (Nordby 1999, 9). Second, academics can be expected to have deeper theoretical knowledge, be more updated on the state of the art and have closer ties to the international discipline. As such, they bring different competences and points of reference to policy formulation.

Beyond the participation of academic actors on advisory commissions, was advice also increasingly based on scientific knowledge and claims? Some interesting patterns can be discerned from the citation analysis. The first is the growing tendency of advisory commissions to support arguments with explicit references. This may be interpreted as a sign of scientization. Explicit referencing is central to the academic enterprise, and the explosion of citations may be seen as evidence that commissions adopted a more scientific style of argumentation. On the one hand, this may be a function of growing expectations that policy arguments are backed up with some form of knowledge or evidence (Weingart 1999; Campbell and Pedersen 2014, 203). On the other hand, it may reflect efforts to present policy advice as “scientific” in order to increase its credibility and authority. Framing advice as scientific, and therefore neutral and objective, may be a strategy to render it immune to political argumentation (Marcussen 2006).

The citation analysis also shows that academic knowledge—almost exclusively from economics—accounted for the largest share of citations in advisory reports. However, while the absolute number of citations to academic economic knowledge increased sharply, their share of total citations was relatively stable. Significant shares of citations went to policy documents expressing political and administrative concerns and to more applied research. In other words, judging from the citation patterns, the knowledge basis of the advisory bodies remained mixed. Academic economic knowledge was an important source of authoritative arguments about policy, but it was not the only one.

A more striking result is the extensive and increasing use of citations to high-prestige, international economic knowledge in commission reports. The orientation towards the most élite international academic journals is remarkable given that these commissions were national policy advisory bodies with mixed membership, whose reports were destined for decision-makers. Yet, this outcome can be interpreted as a result of particular disciplinary dynamics at work. In the highly transnational and hierarchical economics discipline, top international journals constitute the undisputed source of authoritative knowledge (Fourcade et al. 2015). The citation patterns show a clear orientation towards the top of this hierarchy. The strong relationship between citations to international economics journals and the share of academic economists among commission members indicates that economists on commissions upheld strict disciplinary standards about what constitutes knowledge in economic matters. Moreover, the frequent citing of top journals may suggest that links to the international discipline were used as weapons in local struggles over who had authoritative knowledge in thus particular policy area (Dezalay and Garth 2002; Fourcade 2006). One can object that these citations patterns simply reflected the internationalization of the economics profession and had little bearing on the content of policy advice. However, the orientation towards top international journals also entailed a shift in the substance of economic knowledge, towards US-style neoclassical economics characterized by a high degree of formalization and greater attention to micro-economic questions (Fourcade 2009, 2). That this shift had an impact on the content of policy advice is likely and has also been shown empirically in qualitative work (Christensen 2017).

How can these results be interpreted in light of the literature on changes in policy advisory systems? It is possible to see the empirical trends as an expression of externalization, that is, that policy advice increasingly is provided from outside government. An advisory commission is in itself an external advisory mechanism, given that the examination of policy is carried out outside the permanent bureaucracy. (The overall use of advisory commissions in economic policy did not increase over time, though.) In addition, the participation of external actors from academia on these bodies increased. And the commissions increasingly drew on knowledge from outside government, notably international economic research. Yet, the notion of externalization does not capture an essential aspect of the empirical trends, namely the turn towards a specific type of external actors and knowledge.

There are also possible indications of the politicization of policy advice, in the form of the political use of scientific knowledge (Weiss 1979). This especially concerns the growing tendency to appointment academic economists as commission chairmen. The finding that commissions with academic chairmen were no more likely to cite international economic literature than other commissions suggests that scientific experts were drawn upon not only to inform decisions but also to legitimize policy-making. The appointment of economics professors to lead commissions can be seen as a way to signal that policy-making is objective and knowledge-based, thereby bolstering the credibility of policies (cf. Markoff and Montecinos 1993). However, politicization can only describe parts of the observed developments. The broader turn towards academic members—who are presumably animated by scientific ideals of independence and objectivity—runs counter to the idea of more partisan advice. The strong relationship between the share of economists on a commission and citations to economic literature indicates that economists indeed contributed their specialized knowledge to the analysis of policy problems rather than serving as figureheads. The surge in explicit referencing, especially of economic research, is also difficult to reconcile with more politicized advice. (To be sure, commissions may be politicized in ways that are not captured by our data.)

Arguably, the proposed notion of scientization of policy advice better captures the observed trends. The changes in appointments and citations point to a growing tendency of decision-makers to turn to academic experts and expertise for advice on major policy issues. Policy advice provided to government through ad hoc commissions was increasingly supplied by academic actors, rooted in academic knowledge and justified in scientific terms. The broader theoretical implications of these findings are explored in the conclusion.

## Conclusion

This article has shed light on the changing role of academic economic knowledge in policy advisory bodies. Based on a quantitative analysis of Norwegian advisory commissions in economic policy, it has found a growing reliance on academic economists and economic knowledge and an increasing orientation towards economic research from highly prestigious international outlets. This development can be interpreted as a scientization of policy advice in the economic field, that is, a growing reliance on academic expertise for analysis and arguments about public policy.

The article extends scholarly work on the political power of economists into the domain of policy advice. So far, this literature has not explored the implications of the growing role of economists for debates in public policy and administration. Yet, the theoretical argument and findings of the article highlight the relevance of professional factors and dynamics for discussions about policy advice and policy formulation. The findings show that the distinct transnational and hierarchical dynamics of the economics discipline reached far into national policy advisory institutions. This adds to recent studies that have pointed to similar dynamics in other policy-making settings, such as national bureaucracies (Fourcade 2009; Christensen 2017) and international organizations (Chwieroth 2009). It also goes beyond recent work on national regimes of policy research organizations in the economic area by highlighting the role of academic knowledge within these institutions (Campbell and Pedersen 2014).

The article also contributes to the literature on policy advisory systems. While the existing literature presents externalization and politicization as the dominant patterns of change in policy advice (Craft and Howlett 2013), the article points to the scientization of policy advice as a third important trend. The notion of scientization provides a useful addition to the existing literature on policy advisory systems. It shifts the discussion from questions about the location of advice and the degree of governmental control over advice (Craft and Halligan 2017) to questions regarding the epistemic basis for advice: To what extent is policy advice rooted in academic knowledge and justified in scientific terms? How is academic knowledge used in different policy advice institutions? What types of academic knowledge are employed, and to what effect?

The findings from the Norwegian setting also speak to recent debates about the dynamics of policy advice outside the Westminster systems (cf. Van den Berg 2017). In Norway, economic academic knowledge broke through within policy advice bodies despite the pronounced neo-corporatist and statist features of the political system. This points to the limits of broad distinctions such as adversarial versus consensus-based systems for understanding dynamics of policy advice. For instance, countries with extensive neo-corporatist interest representation may also have strong technocratic features, as is the case in many Northern European countries. Policy advice institutions are therefore often hybrid bodies that incorporate different governing logics, such as representative or epistemic logics (Krick 2015). To understand changes in policy advice, it is necessary to examine the interaction between these logics. For instance, how does interest representation change in the face of growing demands for evidence in policy-making?

To be sure, there are important limits to what this analysis can tell us about the changing role of academic expertise in policy advice. The analysis is restricted to a particular type of academic knowledge (economics) and policy area (economic policy). The findings may not be generalizable to other fields. First of all, economic policy may be seen as highly amenable to scientific knowledge, since it addresses a set of complex and uncertain relationships. While significant, this is not unique to economic policy: many modern-day policy issues involve considerable complexity and uncertainty, such as climate change or chemical regulation (cf. Haas 1992). Second, it is often argued that a special relationship has developed between economics and the exercise of state power since World War II (Fourcade 2006, 2009). The tightening of the links between disciplinary knowledge and policy advice that we observe in the economic field may thus be weaker or absent in other fields. Third, the economics discipline has some particular characteristics, such as the transnational and hierarchical character of the field (Fourcade 2006). The shift towards international academic knowledge in policy advice is likely to be weaker for disciplines that have remained more nationally oriented, such as law. Examining whether scientization of policy advice is limited to specific types of academic knowledge or constitutes a more general trend is therefore an important task for future research.

Another limit is that the analysis focuses on a specific advisory institution. It does not capture changes in other parts of the policy advisory system, such as in ministerial advice or in the use of consultants. Developments in these parts of the system may well run counter to the trends observed in the commission system. However, given the central role of ad hoc advisory commissions in the decision-making process on major policy issues, the observed trend is arguably important in and of itself. On a more methodological note, analysing specific advisory institutions rather than the whole system also allows for more precise analyses of change than offered in the existing policy advisory systems literature.

The “million dollar question” is of course what effect the changes in policy advice had on actual policies (Hustedt and Veit 2017, 46). Did the shift towards economic knowledge in advisory commissions give economists greater policy influence? There are reasons why this may not be the case. For instance, highly academic reports may be detached from the real problems of policy-makers and thus stand a smaller chance of being put into practice. Academics may also be called upon to examine policy issues of minor importance, while being sidelined when it comes to politically salient topics (Radaelli 1999). On the other hand, highly academic reports may have a greater chance of being adopted, since they bring innovative solutions to the table rather than rehashing the entrenched positions of government departments and interest groups. (This is an argument frequently heard when speaking to policy-makers.) Ultimately, this is a question that needs to be resolved by systematic empirical analyses of the determinants of the impact of policy advice.

## Appendix 1: Data and coding

The analysis is based on full-text versions of reports from Norwegian Official Commissions (NOU reports) collected from the websites of the National Library of Norway (www.nb.no) and the Norwegian government (www.regjeringen.no). The analysis includes all commissions appointed during the period 1967–2013 that published an NOU report, that submitted their report to the Ministry of Finance, and that had a policy-preparing function. Having a policy-preparing function means that the commission was charged with examining policy questions. This excludes reports from law-drafting commissions (*lovutvalg*). The rationale for this exclusion is the theoretical focus of the article on the role of economic knowledge in substantive policy discussions. Also excluded from the analysis are five reports that were unrelated to economic policy or for which data is missing. In the analysis, the unit of observation is the commission report. Commissions that produced multiple reports are thus counted multiple times. This concerns four commissions, which produced a total of 13 reports. All these reports are included since they often concerned separate issues and were written by a slightly different group of members.

NOU reports are official documents and are recognized as a legal source in Norwegian jurisprudence. Reports normally include information on the chairman and members appointed to the commission, the work of the commission, its considerations and proposals and references to relevant documents and literature.

Appointments to the commissions are coded as follows: “Members” are identified as all members of the commission at the time of appointment plus members who were appointed to the commission later and did not replace existing members. The affiliation of members and chairmen is coded primarily based on the organization or job title given in the reports, which indicates in which capacity the members participate in the commission. “Academic economists” are identified as anyone working in a scientific position in an economics department at a university or university college or anyone working at a research institute whose highest academic degree is in economics. “Civil servants” are identified as administrators in public organizations at all levels. “Politicians” are identified as anyone who at the time of appointment was a minister, undersecretary of state, member or deputy member of parliament or mayor. Where information in the report is insufficient, other sources are used to establish affiliation.

The citation analysis includes all references that are listed either in a bibliography or in footnotes in the commission reports. Multiple citations to the same document in one report are excluded. Citations are coded according to the following scheme (Table 3).

**Table 3:** Coding scheme for the citation analysis

	Policy documents	Policy research	Academic research
National	NOU reports, government bills and acts, laws, etc.	Research from national government bodies, studies commissioned for government reports^a^	Research from Norwegian academic journals, books, etc.
International	Reports, acts from foreign governments and international organizations	Research from foreign government bodies and international organizations	Research from international academic journals, books, etc.

^a^Publications from interest groups, think tanks and consultancy firms are coded as a separate category

For the analysis of journal citations, “economics journals” are identified as journals listed in the economics category of the Thomson Reuters Journal Citations Report 2014 or unlisted journals that clearly belong to the field of economics.

## Appendix 2: Descriptive statistics and correlations

See Tables 4 and 5.

**Table 4:** Descriptive statistics for all variables in the regression analysis

	N	Mean	SD	Min	Max
Citations to international economics journals (log)	79	0.804	1.142	0	3.925
Share of members academic economists	79	0.138	0.178	0	0.75
Chairman academic economist	79	0.304	0.463	0	1
Share of members statistics bureau	79	0.059	0.086	0	0.333
Chairman statistics bureau	79	0.139	0.348	0	1
Year appointed (1967 = 0)	79	23.089	11.845	0	46

**Table 5:** Correlations for all variables in the analysis

	(1)	(2)	(3)	(4)	(5)	(6)
(1) Citations to international economics journals (log)	1.000					
(2) Share of members academic economists	0.636	1.000				
(3) Chairman academic economist	0.097	− 0.110	1.000			
(4) Share of members statistics bureau	0.254	0.302	0.341	1.000		
(5) Chairman statistics bureau	0.396	0.372	− 0.266	− 0.091	1.000	
(6) Year appointed (1967 = 0)	0.553	0.312	0.273	0.219	0.193	1.000

## Footnotes

1 The pattern is similar also if chairmen are excluded from the count, with the share of academic economists rising from 8% in the 1970s to 18% in the 2000s.

2 The correlation (Pearson’s *R*) is − 0,110 and is not statistically significant at the 0.05 level.

3 Since the distribution of the values on the variable is skewed, the variable is log-transformed using log(*x* + 1).
